# Evaluation of developmental competence of Sus scrofa domesticus (L.) oocyte-cumulus complexes after intra- and extraovarian vitrif ication

**DOI:** 10.18699/VJ21.069

**Published:** 2021-10

**Authors:** Т.I. Kuzmina, I.V. Chistyakova

**Affiliations:** All-Russian Research Institute of Genetics and Breeding of Farm Animals – Branch of L.K. Ernst Federal Research Center for Animal Husbandry, Pushkin, St. Petersburg, Russia; All-Russian Research Institute of Genetics and Breeding of Farm Animals – Branch of L.K. Ernst Federal Research Center for Animal Husbandry, Pushkin, St. Petersburg, Russia

**Keywords:** oocyte, vitrif ication, extraovarian, intraovarian, mitochondria, Sus scrofa domesticus (L.), ооцит, витрификация, экстраовариальная, интраовариальная, митохондрии, Sus scrofa domesticus (L.)

## Abstract

The aim of the present study was to identify the inf luence of extra- (EOV) and intraovarian vitrif ication (IOV) on mitochondrial activity (MA) and chromatin state in porcine oocytes during maturation in vitro. During EOV porcine oocytes were exposed in cryoprotective solutions (CPS): CPS-1 – 0.7 M dimethyl sulfoxide (DMSO) + 0.9 M ethylene glycol (EG); CPS-2 – 1.4 M DMSO + 1.8 M EG; CPS-3 – 2.8 M DMSO + 3.6 M EG + 0.65 M trehalose. At IOV the ovarian fragments were exposed in CPS-1 – 7.5 % EG + 7.5 % DMSO, then in CPS-2 – 15 % EG, 15 % DMSO and 0.5 M sucrose. Straws with oocytes and ovarian fragments were plunged into LN2 and stored. For devitrif ication, the EOV oocytes were washed in solutions of 0.25, 0.19 and 0.125 M of trehalose, the IOV – in 0.5 and 0.25 М trehalose. Oocytes were cultured in NCSU-23 medium with 10 % f luid of follicles, follicular walls, hormones. 0.001 % of highly dispersed silica nanoparticles (ICP named after A.A. Chuyko of the NAS of Ukraine) were added to all media. The methods of fertilization and embryo culture are presented in the guidelines developed by us. MA and chromatin state were measured by MitoTracker Orange CMTMRos and the cytogenetic method. Signif icant differences in the level of oocytes with high-expanded cumulus between control and experimental vitrif ied groups (81 % versus 59 % and 52 %, respectively, p ≤ 0.001) were observed. The percentage of pyknotic cells in native oocytes was 19 %, EOV or IOV oocytes were 39 % and 49 %, respectively. After culture, the level of matured native oocytes was 86 %, 48 % EOV and 33 % IOV cells f inished the maturation ( p ≤ 0.001). Differences were also observed in the level of MA between groups treated by EOV and IOV (89.4 ± 7.5 μA and 149.2 ± 11.3 μA, respectively, p ≤ 0.05). For the f irst time, pre-implantation embryos were obtained from oocytes treated by IOV.

## Abstract (ru)

Цель настоящей работы – идентификация влияния экстра- (ЭОВ) и интраовариальной витрификации (ИОВ) на митохондриальную активность (МА), состояния хроматина в ооцитах свиней в процессе созревания in vitro. При ЭОВ ооциты свиней обрабатывали растворами криопротекторов (КПР): КПР-1 – 0.7 M диметилсульфоксида (ДМСО) + 0.9 M этиленгликоля (ЭГ); КПР-2 – 1.4 M ДМСО + 1.8 M ЭГ; КПР-3 – 2.8 M ДМСО + 3.6 M ЭГ + 0.65 M трегалозы. При ИОВ фрагменты яичников опускали в КПР-1 – 7.5 % ЭГ + 7.5 % ДМСО, затем в КПР-2 – 15 % ЭГ, 15 % ДМСО и 0.5 М сахарозы. Пайеты с ооцитами и фрагменты яичников погружали и хранили в LN2. Для девитрификации ЭОВ ооциты экспонировали в 0.25, 0.19 и 0.125 М растворах трегалозы, ИОВ – в 0.5 и 0.25 М трегалозы. Ооциты культивировали в среде NCSU-23 с 10 % жидкости фолликулов, их стенками, гормонами. Все среды дополняли 0.001 % наночастиц высокодисперсного кремнезема (Институт химии поверхности им. А.А. Чуйко Национальной академии наук Украины, Украина). Режимы оплодотворения и культивирования эмбрионов представлены нами в методических рекомендациях. Митохондриальную активность и статус хроматина оценивали MitoTracker Orange CMTMRos и цитогенетическим методом. Выявлены достоверные различия в уровне ооцитов c высокоэкспандированным кумулюсом между контрольной и витрифицированными группами (81 % против 59 и 52 % соответственно, p ≤ 0.001). Доля пикнотических клеток у нативных ооцитов составила 19 %, у ЭОВ и ИОВ ооцитов – 39 и 49 % соответственно. Стадии ме- тафазы II достигли 86 % нативных ооцитов, и только 48 % ЭОВ и 33 % ИОВ ооцитов завершили созревание (р ≤ 0.001). Отмечена достоверная разница в МА между группами, подвергнутыми ИОВ и ЭОВ (89.4 ± 7.5 и 149.2 ± 11.3 мкА соответственно, р < 0.05). Впервые получены доимлантационные эмбрионы из ооцитов сви- ней, подвергнутых интраовариальной витрификации.

## Introduction

The development of a vitrification method for cryopreservation of reproductive cells is the most significant achievement for human and animal ART over the past 70 years (Coello et al., 2018). However, after more than half a century of research in this area, the results for production of viable embryos from devitrified oocytes remain controversial (Mullen, Fahy, 2012). Firstly, this is closely related to the slow-growing progress in upgrading the protocols (parameters) of extra- or intraovarian freezing/thawing technology (Yurchuk et al., 2018).

In case of extraovarian vitrification of female gametes using open freezing systems, such as straws, cryotopes, cryolopes, the saturation of cells by cryoprotectants can be achieved in a short time with a relatively short exposure time in vitrification solutions, as well as the transition of cells to a vitrified state; in case of a closed intraovarian (intrafollicular) system, the exposure time in cryoprotectant solutions significantly increases, and the rate of transition of intracellular water to the “glass-like” phase is slower due to an increase in the eutectic point (Obata et al., 2018). Due to the lengthening of the water phase transition, there is a danger of the formation of extraand intracellular ice crystals, which have a damaging effect on cells (Amstislavsky et al., 2015). However, when using an open method of vitrification, there is a risk of invasion of the vitrification medium and oocyte-cumulus complexes, which can subsequently affect the competence of cells for fertilization and subsequent embryo development (Joaquim et al., 2017). The intraovarian vitrification can become an alternative closed system, which eliminates the damaging effect of resistant cryogenic microorganisms and fungi on ovarian tissue and oocytes (Bielanski, 2012). Meanwhile, the usage of both vitrification models implies the occurrence of temperature- and osmotically-dependent damage to the subcellular compartments of germ and somatic cells (Buderatska, Petrushko, 2016).

The most sensitive organelles are the cytoskeleton, mitochondria, and the nuclear apparatus, which play an important role in the proliferation of somatic cells, as well as the maturation and further development of female gametes (Lai et al., 2014). As a consequence of cryogenic phase-structural transitions and peroxidation of annular lipids, the barrier properties of the mitochondrial membrane are disrupted, there is a leakage of transported ions, including Ca2+ and H+, and metabolites both through the active transport and the passive diffusion via the transmembrane defects (non-specific pores with high permeability), which causes a decrease in the energy supply of the oocyte during development and contributes to triggering apoptosis (Kuzmina et al., 2019). Low-temperature damage to the nuclear apparatus of oocytes is characterized mainly by a decrease in their matrix activity (synthesis of DNA and RNA) due to the cryodenaturation and the loss of enzyme functional activity (Pereira et al., 2019).

Thus, the creation of an optimal and efficient vitrification technology, which would be able to preserve the architectonics and functional activity of cell compartments that ensure the formation of egg competent for fertilization, is one of the main challenges facing reproductive biologists and cryobiologists dealing with the low-temperature preservation of gametes.

The aim of this study is to identify the effect of various models (extra- and intraovarian) of vitrification on the functional activity of mitochondria (fluorescence intensity of MitoTracker Orange CMTMRos) and chromatin status in native and devitrified oocytes Sus scrofa domesticus (L.) during the extracorporeal maturation and the development of preimplantation embryos

## Materials and methods

All reagents used in the experiments, except those indicated in the text, were manufactured by Sigma-Aldrich (USA). Plastic laboratory glassware was from BD Falcon™ (USA).

In the experiments, the oocyte-cumulus complexes (OCC) aspirated from the ovarian antral follicles of S. scrofa domesticus (L.) (domestic pig) of Landrace breed were used. After ovariectomy, the porcine ovaries were delivered to the laboratory in 0.9 % NaCl solution at a temperature of 30–35 °C, containing antibiotics. For the experiments, we used oocytes surrounded by tightly packed layers of cumulus cells (more than five layers), with a uniform zona pellucida, and homogeneous ooplasm. Denuded oocytes and oocytes with loose cumulus were not used.

Cells intended for extraovarian vitrification were treated with three cryoprotectant solutions (CPA) prepared on the basis of TC-199 medium supplemented with 10 % fetal bovine serum (FBS, HyClone, UK): CPA-1 – 0.7 M dimethyl sulfoxide (DMSO) + 0.9 M ethylene glycol (EG); CPA-2 – 1.4 M DMSO + 1.8 M EG; CPA-3 – 2.8 M DMSO + 3.6 M EG + 0.65 M trehalose. Oocyte-cumulus complexes was gradually exposed for 30 sec in CPA-1, then 30 sec in CPA-2 and 20 sec in CPA-3. During intraovarian vitrification, the dissected ovaries were divided into 6–8 sections (15 × 20 mm), placed in sterile gauze bags and dipped in CPA solutions based on Dulbecco’s phosphate buffer solution (PBS) with the addition of 20 % FBS: CPA-1 – 7.5 % EG + 7.5 % DMSO (15 min), then in CPA-2 – 15 % EG, 15 % DMSO and 0.5 M sucrose (2 min). Straws with oocytes and sterile bags with ovarian fragments were immersed in LN2 (−196 °C) for at least 1 h. Extraovarially vitrified OCCs were removed from the straws after thawing and exposed in 0.25 M trehalose solution (3 min) based on medium TC-199 with the supplementation of 10 % FBS at 37 °С, were sequentially washed in a 0.19 M solution (3 min) and then in a 0.125 M solution of trehalose (3 min). Aspirated oocytes from fragments, after thawing, were sequentially treated with 0.5 M (1 min) and 0.25 M (5 min) trehalose solutions prepared on the basis of PBS with 20 % FBS content. The final washing of cells was carried out in TC-199 medium with 10 % FBS. All vitrification/devitrification media were supplemented by highly dispersed silica nanoparticles (nHDS) at a concentration of 0.001 % (Chuiko Institute of Surface Chemistry of National Academy of Sciences of Ukraine, Ukraine). The concentration used in the experiments was chosen according to the data obtained by the developers (Galagan et al., 2010).

Native and devitrified OCCs were cultured in an atmosphere with 5 % CO2 at 90 % humidity, a temperature of 38 °C, in North Carolina State University-23 (NCSU-23) medium with 10 % follicular fluid (from follicles with a diameter of 3–6 mm), 10 M.E. human chorionic gonadotropin, 10 M.E. horse chorionic gonadotropin, fragments of follicular walls (600 × 900 μm), 50 μg/ml gentamicin and 0.001 % HDS nanoparticles (Abeydeera et al., 1998). The protocols of oocyte fertilization and embryo culture are presented in the guidelines (Kuzmina et al., 2008).

For assessing mitochondrial activity in native and devitrified oocytes, a MitoTracker Orange CMTMRos fluorescent probe (Thermofisher Scientific, UK) was used. Oocyte-cumulus complexes was placed into drops of 500 nM solution and incubated in the dark at 37 °C for 30 min. OCCs were washed in PBS with the addition of 0.3 % bovine serum albumin. The washed oocytes were denuded from cumulus cells by incubation in 0.1 % trypsin solution at 37 °С for 5–10 min, transferred into Hanks solution containing 3.7 % paraformaldehyde, and fixed (15 min, 37 °С). After fixation, oocytes were washed in PBS and placed on Superfrost slides.

To analyze the chromatin state, denuded (from cumulus) oocytes and cumulus cells were placed for 5–10 min in a warm 0.9 % hypotonic solution of sodium citrate 3-substituted. Then the cells were fixed on the slides with a mixture of methanol and acetic acid (3:1). Dry-air slides were stained with 4 % Romanovsky–Giemsa solution for 3–4 min (Tarkowski, 1966).

The MitoTracker Orange CMTMRos fluorescence intensity measurement and assessment of nuclear maturation in native and devitrified oocytes, the level of pyknosis in cumulus cells were performed using a fluorescent microscope Axio Imager A2 (Carl Zeiss, Germany) and a photometer (Nikon, Germany). Excitation wavelengths for MitoTracker Orange CMTMRos – 554 nm, radiation – 576 nm. The fluorescence intensity of MitoTracker Orange CMTMRos was measured in μA.

Statistical analysis of the results was carried out using SigmaStat statistical program (Jandel Scientific Software, USA). Results of the present study are predominantly presented using descriptive statistics. Data are presented as means (M) and standard errors (± SEM), as well as frequency variables. To assess the significance of differences between the values, Student’s t-test and Pearson’s χ2 test were used. Results were considered significant when p < 0.05, p <0.01, p <0.001.

## Results and discussion

Difficulties in the development of effective oocyte freezing method are primarily associated with the structural and functional features of the egg organization, as well as the intra- and intercellular signal interactions in devitrified oocytes (Moussa et al., 2014).

**Fig. 1.**
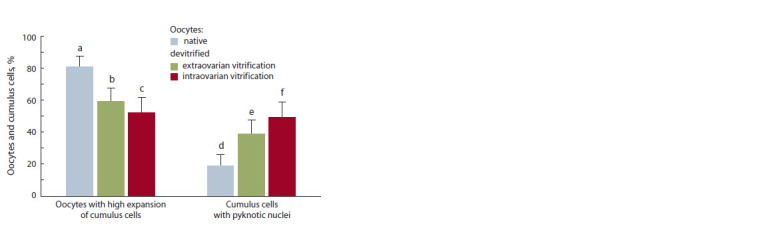
Analysis of cryoresistance indicators of follicular somatic cells (cumulus) of S. scrofa domesticus (L.) with the use of different vitrif ication models (intra- and extraovarian, number of oocytes – 379, number of experiments – 3). Differences are statistically signif icant (χ2-test): a:b; a:c; d:fp ≤ 0.001; d:ep ≤ 0.01.

**Fig. 2.**
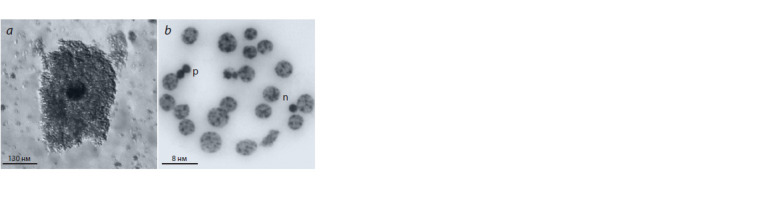
Oocyte-cumulus complex with high cumulus cell expansion (a) and cumulus cells of S. scrofa domesticus (L.) with normal, n, and pyknotic, p nuclei (b) after extraovarian vitrif ication.

In our studies, it was revealed that the proportion of oocytes surrounded by highly-expanded cumulus in the control group significantly exceeded those in devitrified groups, regardless of the vitrification model (81 % versus 59 and 52 %, respectively, p ≤ 0.001) (Fig. 1 and 2). There were no significant differences between the groups of oocytes vitrified outside (extra-) or inside (intra-) fragments of the ovary (see Fig. 1). Analysis of destructive processes in cumulus cells of native and devitrified oocytes showed significant differences in the level of pyknosis between all experimental groups (see Fig. 1). It was found that the native control group had the smallest percentage of chromatin destruction of cumulus cells (19 %). No significant differences were noted in the level of cumulus cells with pyknotic nuclei surrounding extra- and intraovarially devitrified oocytes (39 and 49 %, respectively).

**Fig. 3.**
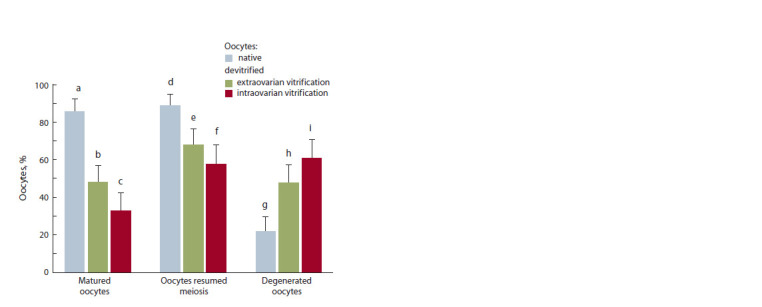
Chromatin status of native and devitrif ied porcine oocytes after in vitro culture with the use of different vitrif ication models (intra- and extraovarian, number of oocytes – 323, number of experiments – 3). Differences are statistically signif icant (χ2-test): a:b; a:c; d:e; d:f; g:h; g:ip ≤ 0.001; b:cp ≤ 0.05.

**Fig. 4.**
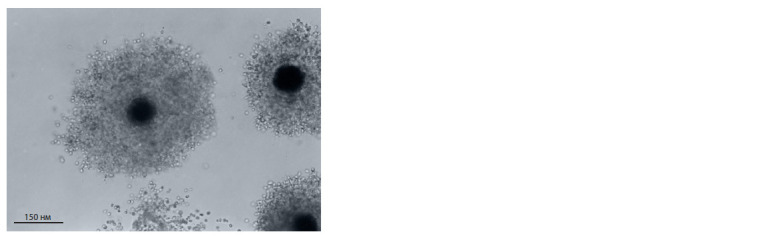
Intraovarially vitrified oocytes of S. scrofa domesticus (L.) after 44 h of in vitro culture.

During in vitro culture of oocytes, it was demonstrated that 68 % of the extraovarilly vitrified cells reinitiated meiosis; in case of intraovarian vitrification, this rate was 58 %, which was significantly lower than that in the native control group (89 %, p ≤ 0.001) (Fig. 3 and 4). About a half of oocytes after extraovarian vitrification (49 %) reached the final maturation stage (metaphase II), the proportion of matured cells previously vitrified within follicles was 33 %, while the percentage of native cells that completed their maturation was 86 % ( p ≤ 0.001). The percentage of cells with the chromatin destruction among native oocytes reached 22 % vs. 48 % and 61 % among extra-/intraovarially vitrified porcine oocytes, respectively ( p ≤ 0.001).

**Fig. 5.**
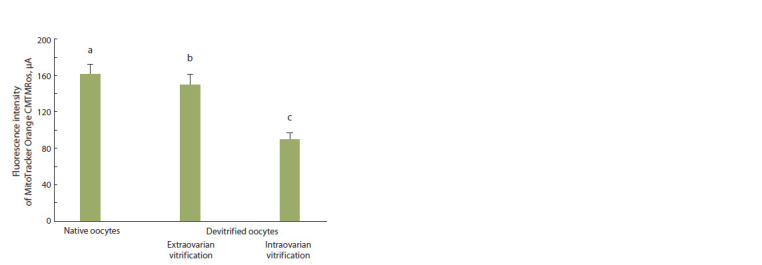
Fluorescence intensity of MitoTracker Orange CMTMRos in native and divitrified oocytes of S. scrofa domesticus (L.) (M ± SEM, number of oocytes – 103, number of experiments – 3). Differences are statistically signif icant (Student-test): a:c; b:cp ≤ 0.05.

**Fig. 6.**
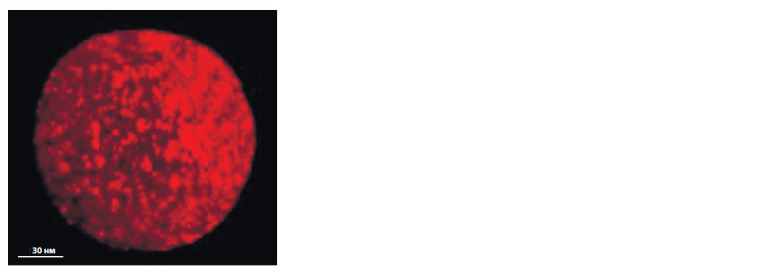
Oocyte of S. scrofa domesticus (L.) with high level of MitoTracker Orange CMTMRos f luorescence intensity after extraovarian vitrif ication.

Mitochondria provide the cell with ATP which is necessary for completion of meiosis, and the features of their functioning are one of the biomarkers of functional state and gamete quality (Al-Zubaidi et al., 2019). It was noted that the mitochondrial potential of intraovarially vitrified oocytes (fluorescence intensity of MitoTracker Orange CMTMRos) was significantly reduced compared to oocytes vitrified outside of the follicles (89.4 ± 7.5 versus 149.2 ± 11.3 μA, respectively, p ≤ 0.05) (Fig. 5 and 6). In the native group of oocytes, the MitoTracker Orange CMTMRos fluorescence intensity was 161.2 ± 10.8 μA.

**Fig. 7.**
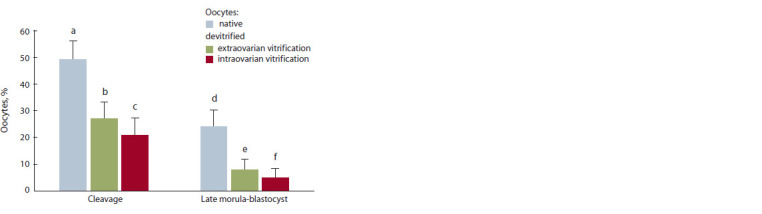
Development of pre-implantation embryos of S. scrofa domesticus (L.) obtained from devitrif ied oocytes (number of oocytes – 556, number of experiments – 3). Differences are statistically signif icant (χ2-test): a:c; d:f p ≤ 0.001; a:b; d:e p ≤ 0.01.

**Fig. 8.**
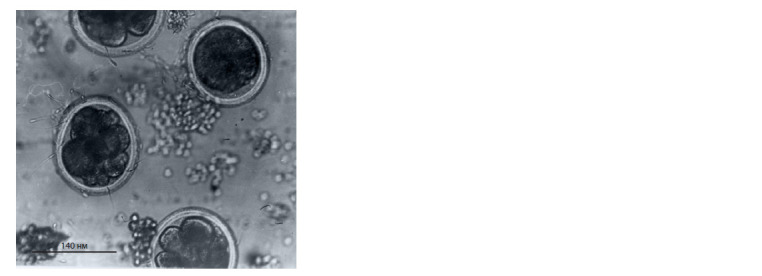
Cleavage embryos obtained from extraovarialy vitrif ied oocytes of S. scrofa domesticus (L.).

Embryos at the final stage of pre-implantation development (the blastocyst stage) were obtained in all experimental groups (Fig. 7 and 8). After fertilization of the experimental groups of oocytes (extra- and intraovarially vitrified), the cleavage rates amounting to 27 and 21 %, respectively, were discovered to be lower than in intact native cells (49 %, p ≤ 0.001). The yield of embryos at late morula, blastocyst stages, obtained from oocytes, vitrified intra- and extraovarially was 5 and 8 %, respectively.

The main indirect indicator by which one can judge the maturity and development competence of the oocyte is the degree of cumulus expansion (Spricigo et al., 2011). According to our analysis of the degree of cumulus expansion after 44 h of culture of native and devitrified oocytes of S. scrofa domesticus (L.), it was demonstrated that the largest proportion of oocytes with a low cumulus expansion was detected among extra- or intraovarially vitrified cells compared to the intact control group (59 and 52 % versus 86 %, p ≤ 0.001). Ultra-low temperatures cause the decrease in the expansion cumulus of porcine oocytes due to the damage of structures called “transzonal bridges” formed by gap junctions and communicating via paracrine signals (Appeltant et al., 2017). The growth of the pyknotic process of the nuclei of devitrified cumulus cells can be explained by the excessive chromosome condensation during the process of cryopreservation (cell dehydration during exposure in cryoprotectant solutions), leading to the “wrinkling” of the cell nucleus, which causes a reduction in the number of normally functioning cumulus cells (Wei et al., 2016; Kokotsaki et al., 2018).

Cumulus cells provide the oocyte with cyclic guanosine monophosphate, which prevents the destruction of cAMP by inhibiting its hydrolysis by PDE3A phosphodiesterase (Mehlmann, 2005), and thus supports the arrest of the first meiotic division at the prophase I stage. With a subsequent decrease in the cAMP level and activation of MPF (maturation promoting factor) due to dephosphorylation of p34cdc2 and the synthesis of cyclin B, the reinitiation of meiosis is stimulated (Yang et al., 2010). Our studies have shown that both models of vitrification (extra- or intraovarian) promote the inhibition of meiosis reinitiation in more than a half of devitrified oocytes (see Fig. 2) due to a thermo-dependent rupture of the communication between cumulus cells and oocyte and, as a consequence, a violation of the concentration balance of intracellular cAMP (Mehlmann, 2005).

During culture of extra- or intraovarially vitrified oocytes, the proportion of matured oocytes with normal chromatin sharply decreases, the level of cells with meiotic aberrations increases compared to the control group, owning to the destruction of nucleotides, the appearance of DNA single-/ double-stranded breaks (Pereira et al., 2019). An increase in the number of degenerated cells during in vitro culture can be associated with the violation of processes of spindle polymerization/ depolymerization during oocyte nuclear maturation (metaphase I–anaphase stage) and subsequent disruption of its assembly, which affects chromosome segregation during the first meiotic division (Yang et al., 2012).

During ultra-low temperature cooling of oocyte, mitochondria are exposed to an excessive load of the ionized form of Ca2+, due to an increase in the cytosolic concentration of Ca2+ in the cell (Shahsavari et al., 2019). Due to such load, non-specific high permeability pores are opened, which leads to the death of oocytes by the apoptotic mechanism (Novoderezhkina et al., 2016). The oxidative stress, mediated by the accumulation of reactive oxygen species, plays the main role in the opening of non-specific pores during freezing, which causes the destruction of membrane proteins and a reduction of the mitochondrial transmembrane potential (Zavodnik, 2016).

Thus, a decrease in the mitochondrial potential during vitrification may be associated with a shift in the concentration of reactive oxygen species, and, as a consequence, an increase in intracellular Ca2+ concentration and the opening of non-specific pores. A significant decrease in mitochondrial functional activity of intraovarially vitrified group of oocytes as compared to extraovarially vitrified oocytes may be caused by additional processes of tissue recrystallization due to insufficient saturation of ovarian tissues with cryoprotectants (Kuzmina, Chistyakova, 2020).

A decrease in the cleavage and embryo yield from intra-/ extraovarially vitrified oocytes is possibly associated with the temporary increase in the intracellular concentration of Ca2+ in gametes during exposure to cryoprotectants and cooling (Larman et al., 2006), which leads to the exocytosis of cortical granules (Kline D., Kline J.T., 1992) and premature hardening of the zona pellucida, which prevents fertilization of the egg.

## Conclusion

Cryobanks, as sources of biological raw materials, are of a great importance for the subsequent use of mammalian oocytes or their ooplasts in cellular and genetic engineering, in particular, the CRISPR-cas9 genomic editing technique, as well as in preserving the gene pool of endangered breeds and genetic diversity. The development of an effective vitrification procedure through different approaches, including the usage of substances of various (natural or synthetic) origin with cryoprotective properties, is the main practical line of development of reproductive biology.

In our work, we analyzed the indicators of nuclear-cytoplasmic maturation of donor porcine oocytes exposed to ultra-low temperatures, including the chromatin state and level of oocyte mitochondrial activity. The revealed features in the functioning of the indicated cell compartments would complement the available data on the nature of destructive processes caused by vitrification/devitrification procedures. Exposure to ultra-low temperatures promoted a decrease in the level of oocytes that completed nuclear maturation and a decrease in MitoTracker Orange CMTMRos fluorescence intensity (a marker of mitochondrial functional activity).

The work also showed the importance of communication between the oocyte and the surrounding somatic cells of the ovarian follicle (cumulus). The cumulus cell morphology after the cryopreservation procedure (vitrification/devitrification) largely determined the “fate” of the oocyte – the completion of nuclear maturation (reaching of the metaphase II stage by the oocyte) and the functional activity of mitochondria. The presented protocols of intra- and extraovarian vitrification/ devitrification, improved by the addition of highly dispersed silica nanoparticles to cryoprotective solutions and culture media, have provided pre-implantation porcine embryos (S. scrofa domesticus (L.)) from oocytes of devitrified ovarian tissue for the first time.

## Conflict of interest

The authors declare no conflict of interest.

## References

Abeydeera L.R., Wang W.H., Cantley T.C., Prather R.S., Day B.N. Presence
of beta-mercaptoethanol can increase the glutathione content
of pig oocytes matured in vitro and the rate of blastocyst development
after in vitro fertilization. Theriogenology. 1998;50:747-756.
DOI 10.1016/s0093-691x(98)00180-0.

Al-Zubaidi U., Liu J., Cinar O., Robker R.L., Adhikari D., Carroll J.
The spatio-temporal dynamics of mitochondrial membrane potential
during oocyte maturation. Mol. Hum. Rep. 2019;25(11):695-705.
DOI 10.1093/molehr/gaz055.

Amstislavsky S.Y., Brusentsev E.Y., Rozhkova I.N., Okotrub K.A.
Embryo and gamete cryopreservation for genetic resources conservation
of laboratory animals. Russ. J. Dev. Biol. 2015;46(2):47-59.
DOI 10.1134/S1062360415020022

Appeltant R., Somfai T., Santos E.C.S., Dang-Nguyen T.Q., Nagai T.,
Kikuchi K. Effects of vitrification of cumulus-enclosed porcine
oocytes at the germinal vesicle stage on cumulus expansion, nuclear
progression and cytoplasmic maturation. Reprod. Fertil. Dev. 2017;
29(12):2419-2429. DOI 10.1071/RD16386.

Bielanski A. A review of the risk of contamination of semen and embryos
during cryopreservation and measures to limit cross-contamination
during banking to prevent disease transmission in ET practices.
Theriogenology. 2012;77(3):467-482. DOI 10.1016/j.theriogenology.
2011.07.043.

Buderatska N.O., Petrushko M.P. Oocytes as alternative to embryos
in cryopreservation applied in assisted reproductive technologies.
Probl.
Cryobiol. Cryomed. 2016;26(4):375-382. DOI 10.15407/
cryo26.04.375.

Coello A., Pellicer A., Cobo A. Vitrification of human oocytes. Minerva
Ginecol. 2018;70(4):415-423. DOI 10.23736/S0026-4784.18.
04218-1.

Galagan N.P., Klymenko N.Y., Orel I.L., Novikova E.A., Turov V.V.
Biofunctional nanomaterials based on ultrafine silica, protein and
aminocarbohydrates. Biopolym. Cell. 2010;26(3):205-213. DOI
10.7124/bc.000158.

Joaquim D.C., Borges E.D., Viana I.G.R., Navarro P.A., Vireque A.A.
Risk of contamination of gametes and embryos during cryopreservation
and measures to prevent cross-contamination. BioMed Res. Int.
2017;4:1-11. DOI 10.1155/2017/1840417.

Kline D., Kline J.T. Repetitive calcium transients and the role of calcium
in exocytosis and cell cycle activation in the mouse egg. Dev.
Biol. 1992;149:80-89. DOI 10.1016/0012-1606(92)90265-i.

Kokotsaki M., Mairhofer M., Schneeberger C., Marschalek J., Pietrowski
D. Impact of vitrification on granulosa cell survival and gene
expression. Cryobiology. 2018;85:73-78. DOI 10.1016/j.cryobiol.
2018.09.006.

Kuzmina T.I., Alm H., Тorner H. Methods of Porcine Embryo Production
in vitro. St. Petersburg, 2008. (in Russian)

Kuzmina T.I., Chistyakova I.V. Assessment of competence for the development
of Bos taurus oocytes after intra- or extraovarial vitrification.
Dostizheniya Nauki i Tekhniki APK = Achievements of Science
and Technology of AIC. 2020;34(2):61-64. DOI 10.24411/ 0235-
2451-2020-10213. (in Russian)

Lai D., Ding J., Smith G.W., Smith G.D., Takayama S. Slow and steady
cell shrinkage reduces osmotic stress in bovine and murine oocyte
and zygote vitrification. Hum. Rep. 2014;30(1):37-45. DOI 10.1093/
humrep/deu284.

Larman M.G., Sheehan C.B., Gardner D.K. Calcium-free vitrification
reduces cryoprotectant-induced zona pellucida hardening and
increases fertilization rates in mouse oocytes. Reproduction. 2006;
131:53-61. DOI 10.1530/rep.1.00878.

Mehlmann L.M. Stops and starts in mammalian oocytes: recent advances
in understanding the regulation of meiotic arrest and oocyte
maturation. Reproduction. 2005;130(6):791-799. DOI 10.1530/rep.
1.00793.

Moussa M., Shu J., Zhang X., Zeng F. Cryopreservation of mammalian
oocytes and embryos: current problems and future perspective.
Sci. China Life Sci. 2014;57(9):903-914. DOI 10.1007/s11427-014-
4689-z.

Mullen S.F., Fahy G.M. A chronologic review of mature oocyte vitrification
research in cattle, pigs, and sheep. Theriogenology. 2012;78:
1709-1719. DOI 10.1016/j.theriogenology.2012.06.008.

Novoderezhkina E.A., Zhivotovsky B.D., Gogvadze V.G. Induction
of unspecific permeabilization of mitochondrial membrane and
its role in cell death. Mol. Biol. 2016;50(1):43-58. DOI 10.1134/
S0026893316010167.

Obata R., Nakumura Y., Okuyama N., Sasaki C. Comparison of residual
dimethyl sulfoxide (DMSO) and ethylene glycol (EG) concentration
in bovine ovarian tissue during warming steps between slow freezing
and vitrification. Cryo Lett. 2018;39(4):251-254. PMID: 30963170.

Pereira B.C., Ortiz I., Dorado J.M., Diaz-Jimenez M.A., Consuegra C.,
Gosalvez J., Hidalgo M. Effect of permeable cryoprotectant-free
vitrification on DNA fragmentation of equine oocyte-cumulus
cells. Reprod. Domest. Anim. 2019;54(3):53-56. DOI 10.1111/rda.
13491.

Shahsavari M.H., Moghaddam G., Daghigh Kia H. Effects of new synthetic
cryoprotectant agents on histological characteristics of various
classes of vitrified bovine pre-antral follicles. Vet. Res. Forum.
2019;10(1):9-16. DOI 10.30466/vrf.2019.34306.

Spricigo J., Rumpf R., Dode M.A.N. Vitrification of bovine oocytes:
effect of the meiotic stage on nuclear and cytoplasmic maturation.
Biol. Reprod. 2011;85(1):721. DOI 10.1093/biolreprod/85.s1.721.

Tarkowski A.K. An air-drying method for chromosomal preparation
from mouse eggs. Cytogenetic. 1966;1:394-400.

Wei J.H., Yuan X.Y., Zhang J.M., Wei J.Q. Caspase activity and oxidative
stress of granulosa cells are associated with the viability
and developmental potential of vitrified immature oocytes. Eur. J.
Obstet.
Gynecol. Reprod. Biol. 2016;198:22-26. DOI 10.1016/
j.ejogrb.2015.12.010.

Yang C.R., Miao D.Q., Zhang Q.H., Guo L., Tong J.S., Wei Y.,
Huang X., Hou Y., Schatten H., Liu Z., Sun Q.Y. Short-term preservation
of porcine oocytes in ambient temperature: novel approaches.
PLoS One. 2010;5(12):e14242. DOI 10.1371/journal.pone.0014242.

Yang C.R., Wei Y., Qi S.T., Chen L., Zhang Q.H., Ma J.Y., Luo Y.B.,
Wang Y.P., Hou Y., Schatten H., Liu Z.H., Sun Q.Y. The G protein
coupled receptor 3 is involved in cAMP and cGMP signaling and
maintenance of meiotic arrest in porcine oocytes. PLoS One. 2012;
7(6):e38807. DOI 10.1371/journal.pone.0038807.

Yurchuk T., Petrushko M., Fuller B. Science of cryopreservation in
reproductive medicine – Embryos and oocytes as exemplars. Early
Hum. Dev. 2018;126:6-9. DOI 10.1016/j.earlhumdev.2018.08.016.

Zavodnik I.B. Mitochondria, calcium homeostasis and calcium signaling.
Biomeditsinskaya Khimiya = Biomedical Chemistry. 2016;
62(3):311-317. DOI 10.18097/PBMC20166203311. (in Russian)

